# Physical Activity Patterns Among Women and Men During Pregnancy and 8 Months Postpartum Compared to Pre-pregnancy: A Longitudinal Study

**DOI:** 10.3389/fpubh.2019.00294

**Published:** 2019-10-16

**Authors:** Katarina Sjögren Forss, Louise Stjernberg

**Affiliations:** ^1^Department of Care Science, Faculty of Health and Society, Malmö University, Malmö, Sweden; ^2^Department of Health Sciences, The Swedish Red Cross University College, Huddinge, Sweden

**Keywords:** men, physical activity, pre-pregnancy, pregnancy, postpartum, women

## Abstract

**Background:** Realizing the essential prerequisite of regular physical activity (PA) it is essential to have knowledge about how different life change events may influence individual's participation. Many studies have documented that pregnancy and the transition to parenthood are life change events associated with decreased PA among women however, the examination of changes of PA in the male parent during this major life change event has been largely neglected in scientific literature and a significant research gap can be found. In the light of this, this longitudinal study aimed to follow changing PA patterns among women and men during pregnancy and 8 months postpartum compared to pre-pregnancy.

**Methods:** In this study, 123 women and 112 men (partners of the women) that lived in the municipality of Karlskrona, Sweden, were included. Data were collected between 2008 and 2010. The self-reported amount of PA performed outdoors and indoors before pregnancy throughout the entire pregnancy, and 8 months postpartum, were measured.

**Results:** We found similar changes in PA patterns among both women and men during pregnancy and 8 months postpartum when compared to pre-pregnancy. In almost all the activities studied except for walking/strolling, a significant decline was found.

**Conclusions:** Our findings contribute new knowledge about changes in men's PA patterns from pre-pregnancy to pregnancy and postpartum. As couples seem to change activity patterns similarly, it is important to promote family-based PA initiatives and encourage couples to be active together during pregnancy and postpartum.

## Introduction

Realizing the essential prerequisite of regular physical activity (PA), particularly in the prevention of chronic disease and in promoting mental health and well-being (1, 2), it is imperative to recognize how different life change events may affect individual's participation in PA. Many studies have documented that pregnancy and the transition to parenthood are life change events associated with decreased PA among women (3–6); however, the examination of changes of PA in the male parent during this major life change event has been largely neglected in scientific literature and a significant research gap can be found.

In a review of parenthood and PA by Bellows-Riecken and Rhodes (5), 25 independent samples were identified, in which a majority (17) focused on women, and seven included both women and men. Only one study focused based only on men. Factors that was found to have a negative impact on participation in PA were for example fatigue, and lack of time, childcare and social support (7–9). Another review (10) focusing on the impact of fatherhood on PA reported that fathers spent less time on moderate to vigorous PA compared to childless men especially when having young children (<6 years). However, from the studies included, it was impossible to conclude the extent to which fatherhood truly impacts PA, as there are no longitudinal studies in the field (10).

Aside from the apparent health benefits of PA for the parents themselves, previous research has shown that parents have a strong influence on the socialization of children in PA (11, 12). This also emphasizes the need to study participation in PA during the life transition that pregnancy and parenthood involves, as it could potentially act as a gateway to a more inactive life, which may also negatively affect the child in the future. Also, little is known about non-intervention changes (amount and type) of PA habits during the transition from pre-pregnancy to pregnancy as well as postpartum, and longitudinal studies in the field are scarce (3). In light of this, the aim of this study was to follow changing PA patterns among women and men during pregnancy and 8 months postpartum compared to pre-pregnancy. Our hypothesis was that pregnancy and parenthood affect PA patterns among women to a greater extent than among men.

## Materials and Methods

### Study Area, Participants, and Design

The study methods are described in detail in two previous papers that have studied parenthood and factors influencing PA and patterns of PA among women and men before and during pregnancy (13, 14). This third paper involving the same study group also adds results from 8 months postpartum. In brief, all pregnant females living in the municipality of Karlskrona, Sweden, who contacted either of the two antenatal clinics in the municipality during March 2008 and February 2009 were asked to participate. Data were collected through self-reported questionnaires at three time points. The first questionnaire (filled in retrospectively) included questions about the situation 1 month before pregnancy and was responded to in accordance with the initial meeting with the midwife. However, the questions concerning PA addressed the 12 months before pregnancy. The second questionnaire (filled in retrospectively) included questions about the entire pregnancy and was responded to 2 months postpartum. The third questionnaire included questions about the situation eight months postpartum and was responded to 8 months postpartum (13, 14). The data collection ended in June 2010. The questions have been applied and validated in the Swedish Survey of Living Conditions (15) and in a national survey about outdoor life and nature tourism in Sweden (16). However, they have not been validated for retrospective use. All three questionnaires included identical questions but did specifically address the time they were supposed to capture the PA for.

The inclusion criteria were pregnant women who understood Swedish. If have had complications, for example a miscarriage, during previous pregnancies or deliveries women were excluded as well as if the midwife did not consider it appropriate to ask about participation due to the woman's general state of health (13, 14).

Participants were asked to mark their outdoor recreational PA from a list of 24 activities that included a wide range of physical and more sedentary activities, as we wished to include activities with a restorative effect on well-being as well as activities associated with PA. The response alternatives about the performance of PA were described as “never,” “1 time per month,” “2–3 times per month,” “several times per week,” and “every day.” For the analysis, the activities were categorized into seven groups: “Strolling/walking” (including strolling, walking, Nordic walking, golf), “exercising” (including jogging, rollerblading, cycling, orienteering, cross-country riding), “aquatic sports” (including canoeing, diving, swimming), “winter sports” (including cross-country skiing, skating, downhill skiing, tobogganing), “participation in non-strenuous activities” (including hunting, sunbathing, angling, camping, bird-watching, motorboating, sailing), and “gardening.” One open question about “PA performed indoors” (that resulted in a seventh category) was also asked, and based on the participants' responses, this included aerobics, floorball, gymnastics, swimming and weight training. An overall category of PA was calculated in which all categories (despite participation in non-strenuous activities) were included. Participants were included in this category if they participated in PA several times per month to every day during the last 12 months before pregnancy, during the entire pregnancy, and 8 months postpartum.

### Statistical Analysis

In the statistical analyses, the McNemar test was used to compare proportions between groups. Participation in the 24 different forms of outdoor recreational PA and indoor PA was separated into “active” (if a person participated in activities several times (>3 times per month to every day) and “not active” (if a person never participated in activities or up to 2–3 times per month). The 24 different forms were divided in eight categories: physical activity, walking/strolling, exercise, aquatic sports, winter sports, gardening, indoor physical activity, and participation in non-strenuous activities. In these categories, the participant needed to be classified as active in at least one of the activities (non-strenuous activities excluded) in order to be classified as active. IBM SPSS Statistics 22 for Windows (IBM Corporation, Armonk, NY, U.S.) was used for the analysis. *P*-values below 0.05 were considered statistically significant.

### Compliance With Ethical Standards

This study was conducted in compliance with the established ethical guidelines of the Declaration of Helsinki. The Regional Ethical Review Board in Lund, Sweden, gave an advisory statement that this study did not need ethical approval, as sensitive personal data (e.g., health, quality of life, political views and religion) were not collected from the participants, and the participants were not required to sign informed consent. The midwives gave oral and written information about the study and participation was voluntary with the right to withdraw at any time without explanation.

## Results

**Figure 1:**
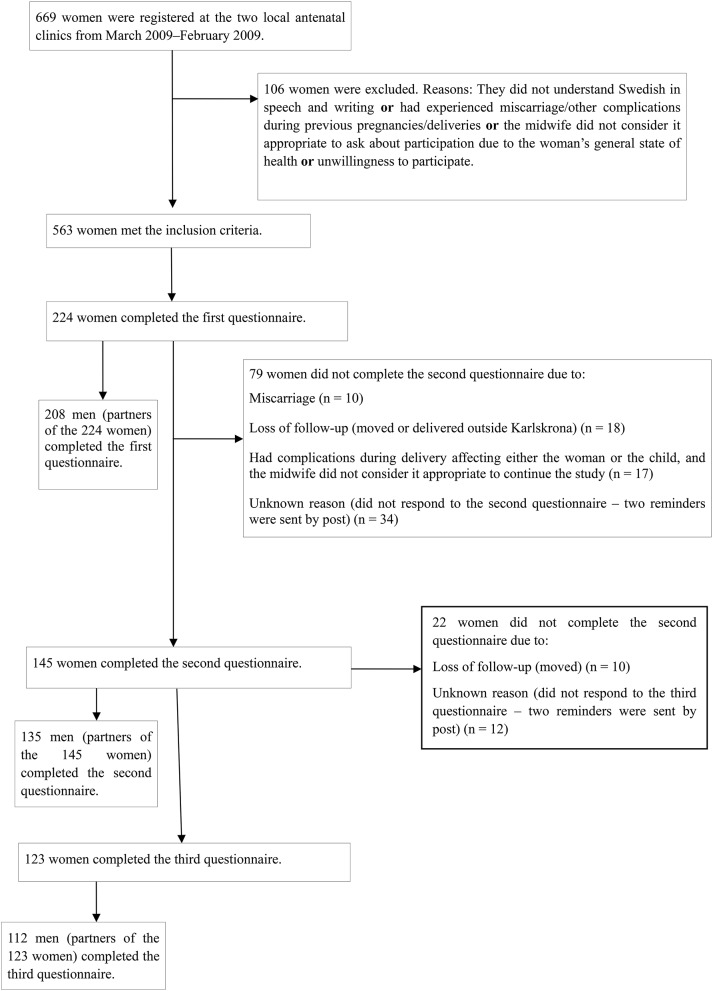
Flow figure chart.

The patterns of PA pre-pregnancy, during pregnancy and 8 months postpartum among 123 women and 112 men (partners of the women) were studied. In Figure 1, a flow chart of participants and dropouts is shown. The response rate at baseline was 40% (224/563), followed by 26% (145/563) at the second data collection and 22% (123/563) at the third data collection. The mean age of the women was 31 years and 33 years for the men. A total of 44% of the women (54/123) and 47% of the men (53/112) were first-time parents. Moreover, 66% of the women (81/123) and 69% of the men (77/112) had a higher education (i.e., completed university education). All those who participated in non-physically strenuous activities were also classed as physically active in at least one of the groups of physical activities.

Among the dropouts, from the first to the second questionnaire, significantly more women (44/79; *p* = 0.006) and men (42/73; *p* = 0.001) had a lower level of education compared to the cohort as a whole. The same could be seen from the second to the third questionnaire, where significantly more women (19/22; *p* = 0.001) and men (18/23; *p* = 0.001) had a lower level of education compared to the cohort as a whole.

### Activity Patterns Among *Women* Pre-pregnancy, During Pregnancy and 8 Months Postpartum

As seen in Table 1, changes in activity patterns among women were almost the same when comparing the situation pre-pregnancy with both during pregnancy and 8 months postpartum. In pre-pregnancy, women did significantly more exercising, aquatic sports, winter sports, PA indoors and non-strenuous activities relative to during pregnancy and postpartum. However, they walked/strolled significantly more during pregnancy and postpartum than pre-pregnancy. No differences in gardening were seen when comparing pre-pregnancy to during pregnancy, but significantly more women did gardening pre-pregnancy than postpartum. Comparing the activity patterns during pregnancy and postpartum, significantly more women performed aquatic sports, gardening, and non-strenuous activities during pregnancy. No significant differences were seen in strolling/walking, exercising, winter sports, and PA indoors 8 months postpartum as compared to during pregnancy. Also, no significant differences were found in the total amount of PA when comparing participation pre-pregnancy to during pregnancy and 8 months postpartum.

**Table 1:** Number of women *n* (%) participating in physical activity before, during, and after pregnancy.

	**Before *n* = 121**	**During *n* = 121**	***p*^a^**	**Before *n* = 121**	**After *n* = 121**	***p*^a^**	**During *n* = 123**	**After *n* = 123**	***p*^a^**
Physical activity^1^	120 (99.2)	118 (97.5)	0.500^b^	120 (99.2)	116 (95.9)	0.219^b^	120 (97.6)	118 (95.9)	0.688^b^
Walking/strolling^2^	95 (78.5)	113 (93.4)	**0.001**	95 (78.5)	111 (91.7)	**0.005**	115 (93.5)	113 (91.9)	0.791^b^
Exercise^3^	81 (66.9)	44 (36.4)	**<0.001**	81 (66.9)	38 (31.4)	**<0.001**	45 (36.6)	39 (31.7)	0.405
Aquatic sports^4^	90 (74.4)	50 (41.3)	**<0.001**	90 (74.4)	31 (25.6)	**<0.001**	51 (41.5)	31 (25.2)	**0.001**
Winter sports^5^	45 (37.2)	23 (19.0)	**<0.001**	45 (37.2)	22 (18.2)	**<0.001**	23 (18.7)	22 (17.9)	1.000^b^
Gardening	63 (52.1)	67 (55.4)	0.597	63 (52.1)	43 (35.5)	**0.003**	67 (54.5)	44 (35.8)	**0.001**
Indoor physical activity^6^	81 (66.9)	45 (37.2)	**<0.001**	81 (66.9)	45 (37.2)	**<0.001**	46 (37.4)	47 (38.2)	1.000
Participation in non-strenuous activities^7^	102 (84.3)	72 (59.5)	**<0.001**	102 (84.3)	45 (37.2)	**<0.001**	74 (60.2)	46 (37.4)	**<0.001**

a *McNemar test, Exact Sig. (2-tailed)*.

b *Binomial distribution used*.

1 *Participants were included in this category if they participated in PA several times per month to every day during the last 12 months before pregnancy, during the entire pregnancy, and 8 months postpartum. All categories (despite participation in non-strenuous activities) were included*.

2 *Strolling, walking, Nordic walking, golf*.

3 *Jogging, rollerblading, biking, orienteering, cross-country riding*.

4 *Canoeing, diving, bathing*.

5 *Cross-country skiing, skating, downhill skiing, tobogganing*.

6 *Floorball, weight training, aerobics, swimming, gymnastics*.

7 *Hunting, sunbathing, angling, camping, bird-watching, motorboating, sailing*.

*Statistically significant p-values showed with bold figures*.

*Different figures are according to some missing values. As they are paired tests, there must be values before and during, before and after, and during and after.

### Activity Patterns Among *Men* Pre-pregnancy, During Pregnancy and 8 Months Postpartum

Men participated in significantly more exercise, aquatic sports, PA indoors, and strenuous activities before pregnancy compared to during pregnancy, but no significant differences were seen in winter sports and gardening (Table 2). During pregnancy and 8 months postpartum, men strolled/walked significantly more than pre-pregnancy. When comparing the activity patterns 8 months postpartum to pre-pregnancy, participation in exercising, aquatic sports, gardening, PA indoors, and in non-strenuous activities was significantly less, and no significant differences were seen in winter sports. Eight months postpartum, no significant differences were seen in strolling/walking, exercising, winter sports, and PA indoors than during pregnancy, but significantly less men participated in aquatic sports, gardening, and non-strenuous activities. No significant differences were seen in the total amount of PA when comparing participation pre-pregnancy to during pregnancy and 8 months postpartum.

**Table 2:** Number of men *n* (%) participating in physical activity before, during, and after pregnancy.

	**Before *n* = 109**	**During *n* = 109**	***p*^a^**	**Before *n* = 109**	**After *n* = 109**	***p*^a^**	**During *n* = 112**	**After *n* = 112**	***p*^a^**
Physical activity^1^	107 (98.2)	105 (96.3)	0.625^b^	107 (98.2)	102 (93.6)	0.180^b^	108 (96.4)	105 (93.8)	0.453^b^
Walking/strolling^2^	68 (62.4)	96 (88.1)	**<0.001**	68 (62.4)	92 (84.8)	**<0.001**	99 (88.4)	95 (84.8)	0.454^b^
Exercise^3^	56 (51.4)	43 (39.4)	**0.019**	56 (51.4)	43 (39.4)	**0.047**	45 (40.2)	45 (40.2)	1.000^b^
Aquatic sports^4^	62 (56.9)	44 (40.4)	**0.011**	62 (56.9)	25 (22.9)	**<0.001**	46 (41.1)	27 (24.1)	**0.003**
Winter sports^5^	17 (15.6)	21 (19.3)	0.557	17 (15.6)	14 (12.8)	0.678^b^	21 (18.8)	14 (12.5)	0.143^b^
Gardening	67 (61.5)	65 (59.6)	0.824^b^	67 (61.5)	43 (39.4)	**0.001**	67 (59.8)	43 (38.4)	**<0.001**
Indoor physical activity^6^	76 (69.7)	63 (57.8)	**0.004^b^**	76 (69.7)	59 (54.1)	**0.002**	64 (57.1)	59 (52.7)	0.458
Participation in non-strenuous activities^7^	79 (72.5)	67 (61.5)	**0.043**	79 (72.5)	41 (37.6)	**<0.001**	69 (61.6)	43 (38.4)	**<0.001**

a *McNemar test, Exact Sig. (2-tailed)*.

b *Binomial distribution used*.

1 *Participants were included in this category if they participated in PA several times per month to every day during the last 12 months before pregnancy, during the entire pregnancy, and 8 months postpartum. All categories (despite participation in non-strenuous activities) were included*.

2 *Strolling, walking, Nordic walking, golf*.

3 *Jogging, rollerblading, biking, orienteering, cross-country riding*.

4 *Canoeing, diving, bathing*.

5 *Cross-country skiing, skating, downhill skiing, tobogganing*.

6 *Floorball, weight training, aerobics, swimming, gymnastics*.

7 *Hunting, sunbathing, angling, camping, bird-watching, motorboating, sailing*.

Statistically significant p-values showed with bold figures.

*Different figures are according to some missing values. As they are paired tests, there must be values before and during, before and after, and during and after.

## Discussion

We found similar trends among both women and men in decreasing frequency of PA during pregnancy and 8 months postpartum as compared to pre-pregnancy, however, overall PA levels did not change. Our findings support our hypothesis, that pregnancy and parenthood affect PA patterns among women to a greater extent than among men.

The 112 women and men of this study lived together, and this may have been a factor in the similar activity patterns. Previous research showed that if the partner were physically active at 18 weeks of gestation, the women also tended to be more active at both 18 and also at 32 weeks of gestation as compared to those who had a less-active partner (17). Other studies (18) have found that partner support during pregnancy may also encourage healthier maternal behavior. In addition, women who experienced more social support, especially from their partner, enhanced their PA during postpartum (19). This could mean that if men are encouraged to remain physically active during the pregnancy period, they can in turn encourage the women to do the same, provided she is healthy. Antenatal classes can be one way to inform men about the importance of their role as a supporter to the woman, with the aim to improve maternal health and well-being during pregnancy and childbirth (20, 21). These classes would also likely be a good arena for providing information about health-promoting strategies concerning PA and to encourage couples to perform PA during pregnancy and postpartum. This could lay the foundation for an active life that may have a positive impact on the children's future PA behaviors through the parents' role-modeling and direct involvement.

Among women, we found a significant decline in almost all activities studied, when comparing pre-pregnancy with both pregnancy and 8 months postpartum. These findings are in concordance with others (3–6) and are not unexpected, as there are both biological (i.e., fatigue), personal (i.e., lack of motivation), and environmental factors (i.e., access to appropriate areas) that influence participation in PA during pregnancy and postpartum (19). Despite this decline in PA, the women still tended to be physically active during pregnancy. Our findings are in concordance with previous studies (22, 23) which show a decrease in participation and/or a shift toward less intense activities later in pregnancy.

The only activity where we found an increase was walking/strolling, where significantly more women participated both during pregnancy and 8 months postpartum compared to pre-pregnancy. This result falls in line with others (6, 24) that found walking to be the most common type of PA during pregnancy. In contrast to our findings, Borodulin et al. (25) found that many women went back to the PA habits they have had before pregnancy already 3 months postpartum, although at a lower intensity. In light of the decline in PA patterns during both pregnancy and postpartum, it is essential to advise and support women whose pregnancy is proceeding normally to regularly engage in PA of moderate intensity for 20 to 30 min per day on most or all days of the week during pregnancy and the postpartum period (1). Findings from van der Pligt et al. (26) show women receiving far less advice about PA during postpartum compared to pregnancy. That might be explained by the fact that PA habits during postpartum is mostly an unknown field (25). Little is also known about changes in PA from pregnancy to postpartum as compared to pre-pregnancy to pregnancy (25). Thus, there is a need for additional research to find effective ways to engage midwives in promoting women's PA during postpartum to encourage women to engage in optimal PA routines for their own health benefits.

From our findings, it was somewhat unexpected to find a significant decline in both exercise and indoor PA among the men during pregnancy as compared to pre-pregnancy. This decline remained 8 months postpartum and indicates that pregnancy and parenthood is a life change event that also has an impact on men's participation in PA. Previous research has shown that parents have a strong impact on children's PA behaviors through role-modeling and direct involvement (11, 12); however, most research focuses on how mothers influence children's PA, and little is known about the role of fathers (27). This, along with the findings from our study, highlights the importance for more research in a field that is still in its infancy. It also highlights that the promotion of PA also should be given to men during the life-changing event that pregnancy and fatherhood means.

### Strengths and Limitations

The findings of this study should be considered in the context of its limitations. It is a small study. Only 22% of the women responded to all three questionnaires which may have influenced the results and thus, generalizing must be done with caution. However, the study is unique both in its longitudinal context and by including the PA patterns of both women and men before pregnancy, during pregnancy and 8 months postpartum. Some couples were not represented by both spouses in the final sample. It can be seen as a possible bias and it can be discussed if the other spouse should have been excluded as well. However, the aim of the study was not to address participation in PA among couples and there might be women living without a partner. Also, we think it can be valuable to demonstrate all answers as longitudinal studies in the field are sparse. PA habits were reported by the participants themselves and this may be a source of misclassification and bias the results. PA measurements using the self-reporting method are not as accurate as they would have been if objective methods were used, such as accelerometers (28). However, self-reports are useful to get information about PA patterns in the population, although there might be a risk that individuals either over or underestimate their answers. If we had used a more extensive questionnaire, e.g., IPAQ (International Physical Activity Questionnaire) it could have given us more evidence about intensity and time. If having this information, probably we could have compared our findings with others. The response alternatives about the performance of PA were described as “never,” “1 time per month,” “2–3 times per month,” “several times per week,” and “every day.” There is a rather high step from 1–3 times per month to several times per month. If a person was participating in PA once a week there was no alternative that represented her/him since she/he was participating more than “2–3 times per month” but less than “several times per week.”

The categorization of “active” and “not active” individuals do not comply with general PA guidelines. We only asked the participants about how often they had participated in PA that also must be seen as a limitation. If we also have asked about the intensity or amount of time spent on the activity, we would have had more information about their habits as well as changes in activity patterns. In order to evaluate if a person is active or not it is also important to study how much time is spent in activities. Another limitation is that we did not study potential confounders that could have given the opportunity to evaluate determinants of change in PA patterns due to pregnancy and postpartum in men and women. We wanted to study frequencies of activity patterns, and thus, we tried to include different activities which might be practiced only part of the year in the questionnaire as the seasons tend to have an impact on what kind of PA individuals perform. Consequently, it might be hard to make comparisons regarding when during a year a questionnaire is sent out. As we included a varied range of activities, we have had the possibility to get information about what kind of PA individuals are engaged in. According to the retrospective nature of the study bias may exist, however, the participants were informed about both the second as well as the third questionnaires at the start of the study even if both questionnaires were done retrospectively.

## Conclusion

The findings from this study contribute new knowledge about changes in men's PA patterns from pre-pregnancy to during pregnancy and postpartum. As couples seem to change activity patterns similarly, it is important to promote family-based PA initiatives and encourage couples to be active together during pregnancy and postpartum. Thus, it is possible to lay a foundation for an active life and establish healthy forms of behavior that could lead to long-term health effects for the whole family. There is a need for more longitudinal as well as qualitative studies to get more knowledge about how pregnancy and parenthood affect participation in PA among both women and men and also if and when they return to PA postpartum and how their activity patterns might have changed.

## Ethics Statement

This study was conducted in compliance with the established ethical guidelines of the Declaration of Helsinki. The Regional Ethical Review Board in Lund, Sweden, gave an advisory statement that this study did not need ethical approval and the participants were not required to sign informed consent. The midwives gave oral and written information about the study to the participants, and participation was voluntary. The participants had the right to withdraw at any time without further explanation.

## Author Contributions

Both authors were responsible for the conception, design, and acquisition of data. KS was responsible for the analysis and interpretation of data and drafting the manuscript. LS was responsible for the critical revision of the paper. Both authors read and approved the final manuscript.

### Conflict of Interest

The authors declare that the research was conducted in the absence of any commercial or financial relationships that could be construed as a potential conflict of interest.
